# Determinants of Poor Treatment Outcomes Among Snakebite Envenoming Patients

**DOI:** 10.5334/aogh.5147

**Published:** 2026-04-10

**Authors:** Narendar Kumar, Azfar Athar Ishaqui, Pushp Lata Rajpoot, Muhammad Bilal Maqsood, Razia Sultana, Muhammad Saleh Khaskheli, Adnan Iqbal, Shahida Tabassum, Shaib Muhammad

**Affiliations:** 1 Department of Pharmacy Practice, Faculty of Pharmacy, University of Sindh, Jamshoro 76080, Pakistan; 2 School of Pharmaceutical Sciences, Universiti Sains Malaysia, Pulau Pinang 11800, Malaysia; 3 Department of Clinical Pharmacy, College of Pharmacy, King Khalid University, Abha 62583, Saudi Arabia; 4 Department of Public Health, College of Nursing and Health Sciences, Jazan University, Jazan 45142, Saudi Arabia; 5 Quality and Performance Administration, Eastern Health Cluster, Health Holding Company, Dammam 32253, Saudi Arabia; 6 Institute of Pharmaceutical Sciences, Peoples University of Medical and Health Sciences for Women, Shaheed Benazirabad, Pakistan; 7 Department of Anesthesiology, SICU and Pain Center, Peoples University of Medical and Health Sciences for Women, Shaheed Benazirabad, Pakistan; 8 Department of Pharmacology, Faculty of Pharmacy and Pharmaceutical Sciences, University of Karachi, Pakistan; 9 Department of Statistics, The Women University Multan, Pakistan; 10 Department of Pharmaceutics, Faculty of Pharmacy, University of Sindh, Jamshoro 76080, Pakistan

**Keywords:** snakebite, treatment outcomes, Pakistan, prospective cohort

## Abstract

*Background:* Snakebite envenoming remains a significant yet neglected public health problem in tropical countries, particularly in rural South Asia. This study aimed to identify demographic characteristics, management practices, and the determinants of poor treatment outcomes among snakebite patients in Sindh, Pakistan.

*Methods:* A prospective cohort study was conducted at Peoples Medical College Hospital (PMCH), Shaheed Benazirabad, Sindh, Pakistan, from July 1, 2023, to June 30, 2024. A non-probability purposive sampling technique was used for data collection, and all consecutive patients presenting with confirmed or suspected snakebite were included. Data were collected through a validated study tool on demographics, pre-hospital management, hospital care, and treatment outcomes. Categorical variables were tested with the Chi-square test, and Kaplan–Meier survival analysis was used to test the effect of exposure-to-reporting time and hospital stay time on outcomes using IBM SPSS V29.

*Results:* A total of 320 patients were included; 74.7% were male, and 98.4% were from rural areas. Most victims were aged 20–29 years (31.9%) and engaged in farming or manual labor (67.2%). Nearly half (49.7%) of the bites occurred during summer. Delayed hospital presentation was common, with 22.8% arriving after six hours of the bite. The overall poor-outcome rate was 10.9%, and mortality was 1.9%. A significant association was found between exposure-to-reporting time (*p* = 0.040) and hospital stay duration (*p* < 0.001) with treatment outcomes.

*Conclusion:* Delayed presentation to the hospital and prolonged hospitalization were major predictors of poor outcomes following snakebite. Strengthening emergency referral systems, ensuring timely antivenom availability, and promoting community awareness are essential to reduce morbidity and mortality in snakebite-endemic regions of Pakistan.

## Introduction

Snakebite envenoming is widely regarded as a serious, but neglected public health problem in many tropical and subtropical regions of the world. According to the World Health Organization (WHO), around 1.8–2.7 million people suffer from snake envenomation annually. These incidents result in 80,000 to 138,000 deaths, as well as three to five times as many permanent disabilities among survivors [1]. The burden rests on the low‑ and middle‑income countries and on rural agricultural communities, as they are exposed through their occupations and have limited access to timely medical care and antivenom. In South Asia, especially Pakistan, snakebite is one of the major causes of morbidity and mortality, but it is under‑reported and poorly characterized [2].

Snakebite outcomes range from complete recovery to permanent disability or death. Several factors affect the prognosis, including delayed care, erroneous pre‑hospital treatment, snake species, venom dose, and complications such as acute kidney injury and coagulopathy [1]. Socio‑economic constraints and lack of awareness often result in reliance on traditional healers, causing further delay in definitive treatment. Although studies from India [3], Bangladesh [4], and Nepal [5] have examined these predictors, few prospective studies have been undertaken in Pakistan [6, 7], where there may be regional differences in the species of snakes and accessibility to healthcare.

Given these gaps, prospective studies are very important to provide context‑specific evidence. This study was carried out in Peoples Medical College Hospital (PMCH), Shaheed Benazirabad, Sindh, Pakistan, a tertiary care center catering to a large catchment area of rural population to identify factors affecting poor treatment outcomes following snakebite envenoming. By measuring demographic, pre‑hospital, and in‑hospital management variables, the aim of this research is to help guide clinical decision‑making, community education, and policy interventions to reduce mortality and enhance recovery in snakebite victims in Pakistan.

## Methodology

### Study design

This was a prospective cohort study on consecutive patients registered in the PMCH.

### Study setting

The study was performed at the emergency department of PMCH, Shaheed Benazirabad, Sindh, Pakistan. Data collection was carried out over a one‑year period, i.e., July 1, 2023–June 30, 2024, using a consecutive sampling technique. An additional period of one month was added for the follow‑up. The patients were recruited from the emergency department and were followed up in the wards and intensive care units (ICUs) and again one month after discharge at home.

### Sampling method

Non‑probability purpose sampling technique was used for the data collection.

### Study participants

At triage, patients were evaluated immediately. Suspected cases of snakebite victims were given first aid and admitted to the medical ward for further treatment. Stable patients were discharged when they improved; critically ill patients were moved to the ICU. The data were collected at all levels of the hospital, and follow‑ups were performed through telephone 30 days after discharge.

#### Inclusion criteria

All patients of all age groups with confirmed or suspected snakebite envenoming who gave informed consent were included in the study.

#### Exclusion criteria

Patients were excluded if they (i) had significant comorbidities, (ii) were dead on arrival, (iii) left against medical advice, or (iv) did not provide consent, or in the case of minors, consent was not provided by their legal guardians.

### Data collection instrument

Data were collected using a predesigned and structured study instrument that was adapted from validated tools used in previous studies [8, 9]. The content validity of the instrument was assessed by three academic experts, and a pilot study was conducted to ensure that the instrument was contextually relevant; pilot data were not included in the final analysis.

### Variables

The study collected data on demographics, pre‑hospital care information, clinical management, and outcomes. The demographic characteristics included gender, age, marital status, residential area, education, profession, season and month of exposure, while pre‑hospital care and clinical management included site of exposure, type of admission, exposure‑to‑reporting time, circulatory support, oxygen support, catheterization, symptomatic treatment, antidote, ICU support, ventilator support, and duration of stay at hospital. Treatment outcomes were determined as:

**Good outcome:** Patients who experienced the exposure to poison and completely recovered from the toxic effects of poisoning in the hospital or within 30 days of being discharged.

**Poor outcome:** Patients who died or did not recover from toxic effects fully during hospitalization or within 30 days after discharged.

### Statistical analysis

Data were originally recorded on paper forms. After coding, data were entered into Microsoft Excel and then exported to IBM SPSS Statistics 29 (IBM Corp. Released 2023. IBM SPSS Statistics for Windows, Version 29.0.2.0 Armonk, NY) for analysis. The categorical variables were presented as frequencies and percentages. Continuous variables were categorized into standard intervals. The chi‑square test was applied to determine associations between treatment outcomes and independent variables. The effect of time on patient outcomes was analyzed using Kaplan–Meier survival curves. A p‑value < 0.05 was considered statistically significant.

### Ethical considerations

Ethical approval was obtained from the Bioethics Committee of the University of Sindh, Jamshoro, Pakistan (Reference No. ORIC/SU/1484). Written informed consent was obtained from all participants or their guardians in accordance with the Declaration of Helsinki.

## Results

### Demographic characteristics of patients

During the study period, 320 cases of snakebite were screened for the final data analysis. Among the study participants, the majority were male (74.7%) and aged 20–29 years (31.9%). Additionally, more than half of the patients (54.7%) were married, and the majority belonged to rural areas (98.4%). Furthermore, a major chunk of patients (71.6%) were uneducated, and farmers/laborers (67.2%) by profession. The study also found that about half of the snakebite incidents (49.7%) occurred in the summer season. Table 1 details the demographics of patients with snakebite incidents.

**Table 1:** Demographic characteristics of snakebite patients.

DEMOGRAPHIC CHARACTERISTIC		*N*	%	TREATMENT OUTCOME		*P*‑VALUE
				POOR	GOOD	
Gender	Female	81	25.3	12	69	0.196
	Male	239	74.7	23	216	
Age group (years)	<12	25	7.8	1	24	—**
	12–19	59	18.4	4	55	
	20–29	102	31.9	8	94	
	30–39	69	21.6	10	59	
	40–49	42	13.1	9	33	
	> = 50	23	7.2	3	20	
Marital status	Married	175	54.7	22	153	0.304
	Unmarried	145	45.3	13	132	
Residential area	Rural	315	98.4	34	281	—**
	Urban	5	1.6	1	4	
Education	Educated	91	28.4	11	80	0.678
	Uneducated	229	71.6	24	205	
Profession	Farmer/laborer	215	67.2	24	191	—**
	Unemployed	64	20.0	9	55	
	Student	30	9.4	1	29	
	Others*	11	3.4	1	10	
Season	Autumn	45	14.1	7	38	—**
	Spring	90	28.1	9	81	
	Summer	159	49.7	18	141	
	Winter	26	8.1	1	25	

*Government job, landlord, private job, retired, self‑employed.

**not fulfilled χ2 test assumption.

### Monthly distribution of snakebite envenoming cases

**Figure 1:**
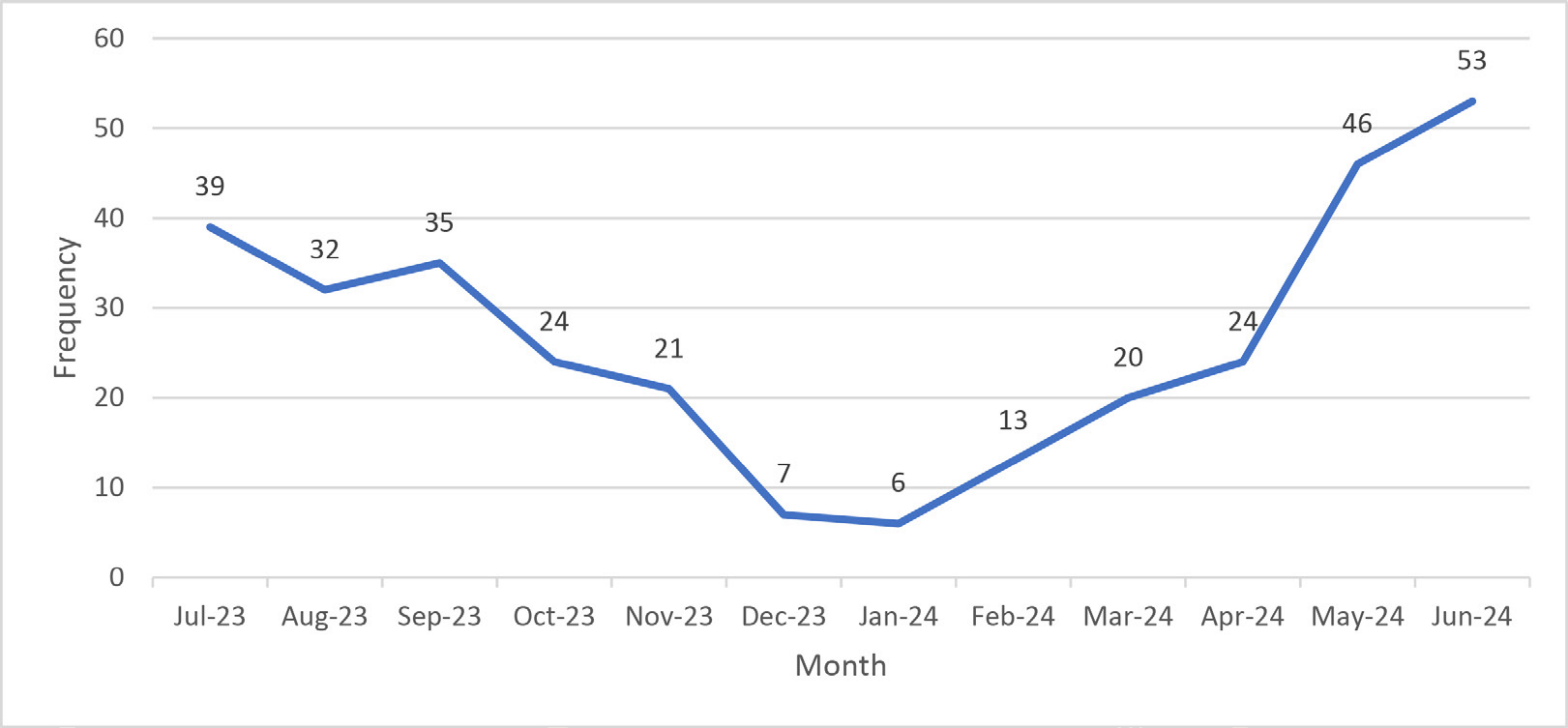
Monthly distribution of snakebite envenoming cases.

Figure 1 details the annual variations in the poisoning incidents. The poisoning incidents peaked during the month of June and then there was a decline in the incidences, with the minimum number of snakebite incidents reported in the month of January.

During the research period, more than half of the incidents (55.3%) were reported from the workplaces. Furthermore, the majority of patients were direct admissions (57.2%) to the study center and arrived after 1–3 hours (43.1%) of snakebite. The mode of transport was mainly non‑ambulance (85.9%).

All the patients of snakebite incidents were given circulatory support (100%), and oxygen support was required by only 4.7%. In the therapeutic interventions, symptomatic treatment (99.4%) was the prevalent treatment, and antidotes were administered to 98.8% of patients. The ICU admission was required for 5.3% patients, and only 1.9% patients required ventilator support. Furthermore, 76.9% of the patients stayed in the hospital for only one day. Table 2 details the patient handling and medical management.

**Table 2:** Patient handling and medical management.

VARIABLE		*N*	%	TREATMENT OUTCOME		*P*‑VALUE
				POOR	GOOD	
Site of exposure	Workplace	177	55.3	19	158	0.939
	Home	131	40.9	15	116	
	Public area	12	3.8	1	11	
Type of admission	Direct admission	183	57.2	16	164	0.713
	Referred	137	42.8	16	121	
Exposure‑to‑reporting time	<1 h	67	20.9	5	62	**0.040**
	1–3 h	138	43.1	10	128	
	3–6 h	42	13.1	6	36	
	>6 h	73	22.8	14	59	
Circulatory support		320	100.0	35	285	—*
Oxygen support		15	4.7	6	9	—*
Catheterization		9	2.8	4	5	—*
Symptomatic treatment		318	99.4	35	283	—*
Antidote		316	98.8	35	281	—*
ICU support		17	5.3	7	10	—*
Ventilator support		6	1.9	3	3	—*
Duration of stay at hospital	1 day	246	76.9	15	231	**<0.001**
	>1 days	74	23.1	20	54	

*not fulfilled χ2 test assumption.

**Figure 2:**
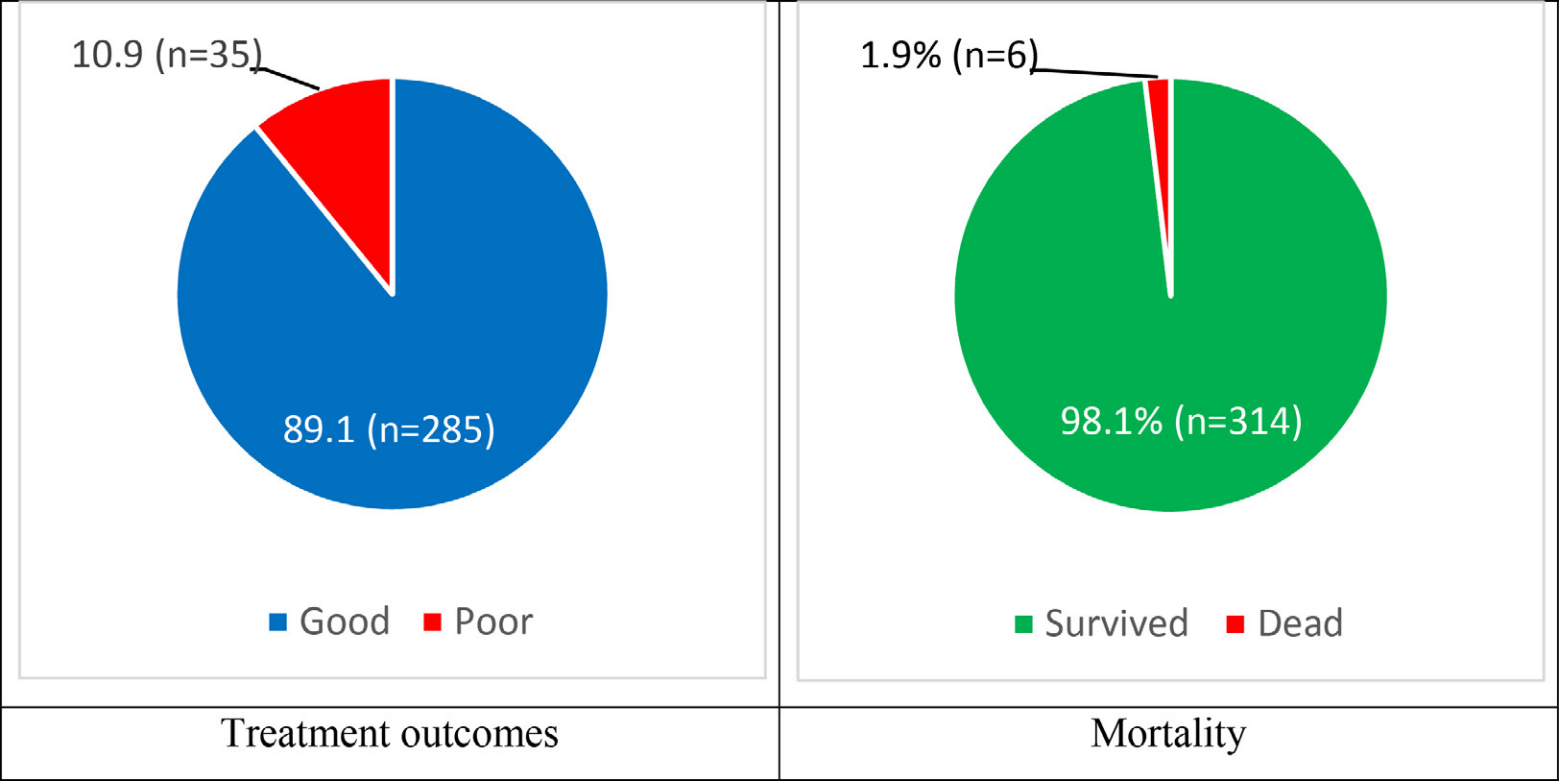
Treatment outcomes of snakebite envenoming patients.

In the outcomes, it was found that 10.9% patients had poor outcomes and 1.9% died because of snakebites (Figure 2).

**Figure 3:**
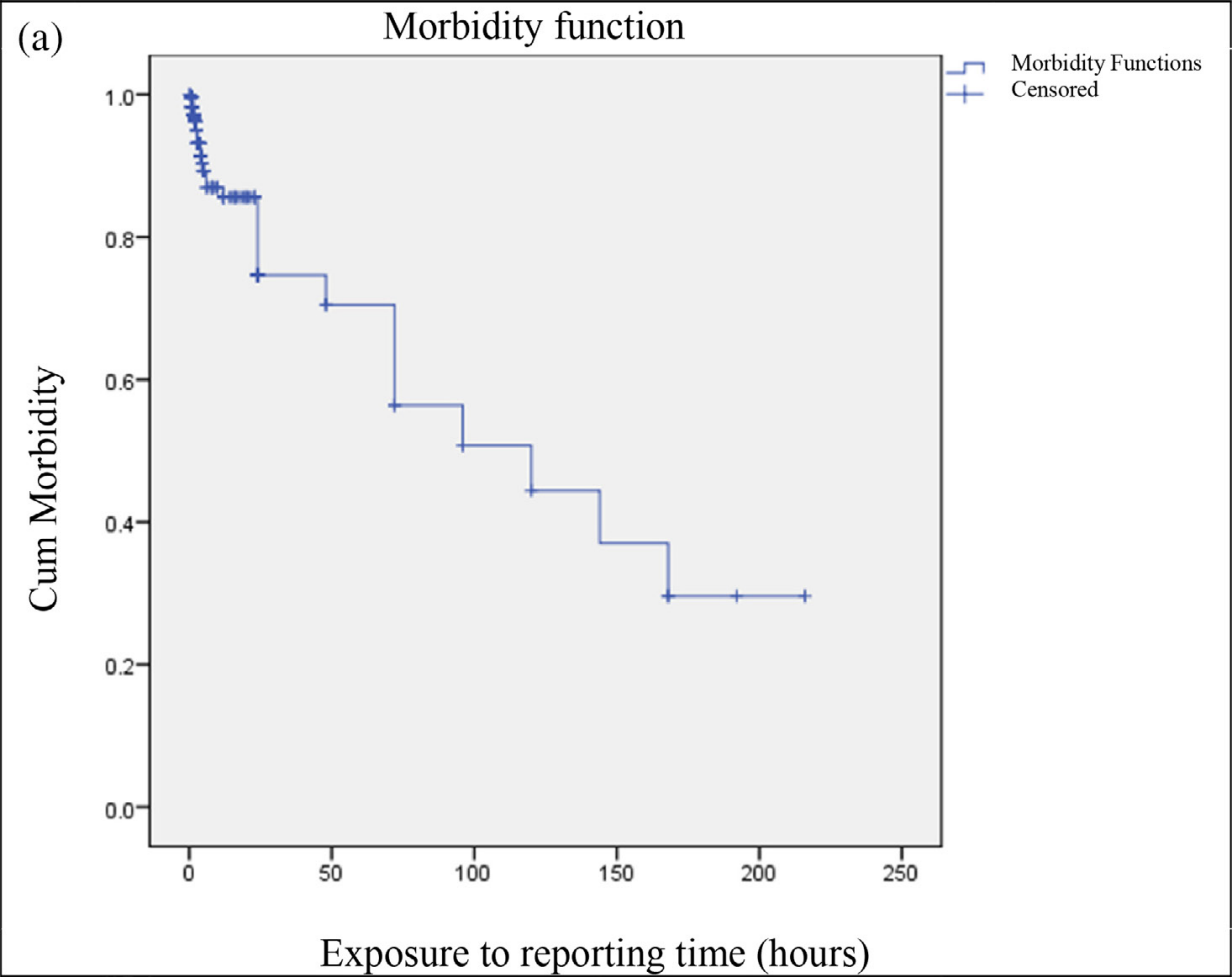

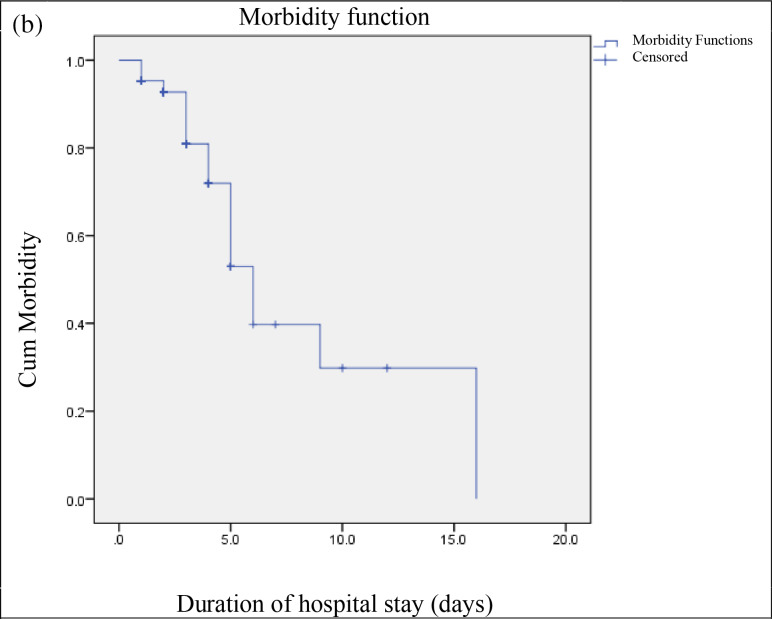
Kaplan–Meier curves for poor treatment outcomes over time. **(a)** Poor outcome vs exposure to reporting time (hours). **(b)** Poor outcome vs duration of stay at hospital (days).

The chi‑square test indicated significant association between exposure‑to‑reporting time (*p* = 0.040) and duration of stay at the hospital (*p* < 0.001). This was further analyzed through Kaplan–Meier curve and association of outcome was related to exposure‑to‑reporting time and duration of stay at hospital. The graphs in Figure 3 indicate that as the time increases the chances of poor outcomes also increase.

## Discussion

This prospective cohort study examined demographic factors, management practices, and predictors of poor outcomes among snakebite envenoming patients in Sindh, Pakistan. The findings demonstrate that most victims were young males from rural areas and engaged in farming or manual labor. This pattern is consistent with studies from India [3, 10], Bangladesh [4], and Nepal [5, 11], where agricultural activity and outdoor exposure substantially increase the risk of snakebite. Similar demographic features have also been described in studies in Pakistan [6, 12], which reflect the occupational nature of exposure in the agrarian area.

The study highlights a seasonal variation in snakebite envenoming, as almost half of the cases occurred in summer and the least number in winter. Comparable seasonal peaks have been reported from India [3, 10] and Bangladesh [4], which may be attributed to both increased agricultural activity and increased snake mobility during warmer months [4]. These outcomes provide emphasis on the need to carry out community awareness and preventive education campaigns prior to high‑risk seasons. This also identifies ready availability of antidotes during these months.

More than half of the incidents occurred in the workplace, and delayed presentation to the hospital was not uncommon: approximately 43% of incidents were reported within 1–3 hours of the bite, and nearly one‑quarter presented more than 6 hours after the bite. The exposure‑to‑reporting interval was significantly associated with poor outcomes (p = 0.040). Similar findings were recorded across South Asia where delays of more than two hours significantly increased morbidity and mortality [3–5]. In Pakistan, lack of emergency transport, reliance on traditional healers, and lack of first aid awareness are still contributing factors to such delays [7]. The predominance of non‑ambulance transport (85.9%) in the present study highlights the need to improve the rural emergency referral systems.

Nearly all patients received antivenom and symptomatic treatment, suggesting that treatment was adequate once patients arrived at the hospital and the tertiary care was in place. The overall mortality rate of 1.9% aligns with the reports from India and Nepal with hospital mortality range of 1–3% [5, 11]. These similarities indicate that good in‑hospital management, such as the availability of antivenom, fluid therapy, and intensive care support, may help to manage the consequences of delayed presentation if they are timely implemented. However, prolonged hospital stay was significantly associated with outcome (p < 0.001), which is likely related to the severity of envenoming and associated complications in delayed presenters. Kaplan–Meier analysis further confirmed the correlation between longer time of exposure‑to‑reporting time and worse prognosis, as was previously reported, and underscores the importance of the “golden hour” of antivenom administration [10].

Socio‑economic and educational inequalities also played a role. Over 70% of patients were uneducated, a situation comparable to that reported from Bangladesh [4] and India [5]. Low literacy is likely to contribute to poor awareness about immediate first aid and the importance of quick referral to a hospital. Community‑based education, training of primary care workers, and dissemination of standardized first aid guidelines to the public could help promote better early response and reduce delays in care‑seeking.

The results confirm that delayed presentation is the single most important determinant of poor treatment outcome. Strengthening ambulance networks, incorporating management of snakebites in existing rural health programs, and providing antivenom consistently are important components of these interventions. Furthermore, setting up regional poison control centers and adopting a framework of follow‑up could improve patient outcomes and enable more accurate reporting of data.

There are a number of strengths to this study. It is one of the few prospective cohort studies of outcomes of snakebites carried out in Pakistan, with systematic data collection at all levels of care and follow‑up. The selection of all consecutive cases over a period of one year reduces the risk of selection bias and increases representativeness. The use of validated data collection tools, expert review, and 30‑day follow‑up of patients after discharge enhances the validity of results, and the use of Kaplan–Meier survival analysis provides analytical depth to assess time‑dependent outcomes. However, there are some limitations that one should be aware of. Being a single‑center study, the results may not be generalizable to other healthcare settings and in particular remote rural areas. Moreover, the study does not provide any information regarding snake species or type of venom due to the absence of diagnostic facility to identify neurotoxic or cytotoxic venom. The classification of outcomes based solely on patient history or visual description alone is prone to substantial misclassification bias, which may limit the strength of related clinical correlations. Additionally, some of the pre‑hospital data were self‑reported by patients or attendants, increasing the potential for recall bias. Lastly, the absence of multivariate binary logistic regression analysis limited the possibility of determining independent predictors of poor outcomes, due to a few factors with p‑values < 0.05 in chi‑square tests. Despite these limitations, the study provides valuable, context‑specific evidence that can be used to guide improvements in snakebite management and future multicenter and interventional research in Pakistan and similar resource‑limited settings.

## Conclusion

This study is one of the few prospective data sets on the outcomes of snakebites in Pakistan. It shows that timely medical attention, appropriate hospital management, and public education are important for the reduction of morbidity and mortality. In areas where snakebites are common, reducing delays in seeking care and improving healthcare systems should be a top public health priority.

## Data Availability

The data will be available upon reasonable request from the authors.

## Financial Declaration

The authors have no other specific funding to declare.

## Authors Declaration

All authors had access to the data and a role in writing the manuscript.
